# Crystallographic and spectroscopic characterization of 5-chloro­pyridine-2,3-di­amine

**DOI:** 10.1107/S2056989017010489

**Published:** 2017-07-21

**Authors:** Aron Sulovari, Joseph M. Tanski

**Affiliations:** a Department of Chemistry, Vassar College, Poughkeepsie, NY 12604, USA

**Keywords:** crystal structure, hydrogen bonding, π-stacking

## Abstract

The two *ortho*-amino groups of the title compound, C_5_H_6_ClN_3_, twist out of the plane of the mol­ecule to minimize intra­molecular inter­action between the amino hydrogen atoms. In the crystal, the amino groups and the pyridine N atom engage in inter­molecular hydrogen bonding. The mol­ecules pack into spiral hydrogen-bonded columns with offset face-to-face π-stacking.

## Abstract 2

The *ortho*-amino groups of the title compound, C_5_H_6_ClN_3_, twist out of the plane of the mol­ecule to minimize intra­molecular inter­action between them. The amino groups and the pyridine N atom engage in inter­molecular hydrogen bonding. The mol­ecules pack into spiral hydrogen-bonded columns with offset face-to-face π-stacking.

## Chemical context   

The title compound, 5-chloro­pyridine-2,3-di­amine, is a tri­sub­stituted pyridine featuring *ortho*-amino groups and a chlorine atom. While all of the sixteen isomers of 5-chloro­pyridine-2,3-di­amine are commercially available, none of their crystal structures have been reported in the literature. 5-Chloro­pyridine-2,3-di­amine may be produced by nitrating 2-amino-5-chloro­pyridine with nitric acid to give 2-amino-3-nitro-5-chloro­pyridine, which is then reduced with sodium di­thio­nite (Israel & Day, 1959 ▸). The reduction may also be accomplished with hydrogen gas and Pd/C (Xie *et al.*, 2016 ▸). 5-Chloro­pyridine-2,3-di­amine has proven useful as a reagent in complex syntheses, such as in the synthesis of aldose reductase inhibitors with anti­oxidant activity (Han *et al.*, 2016 ▸), the regioselective functionalization of imidazo­pyridines *via* alkenylation catalyzed by a Pd/Cu catalyst (Baladi *et al.*, 2016 ▸), the preparation of amino acid oxidase inhibitors (Xie *et al.*, 2016 ▸), the preparation of β-glucuronidase inhibitors (Taha *et al.*, 2016 ▸), the preparation of imidazo­pyridine derivatives with activity against MCF-7 breast adenocarcinoma (Püsküllü *et al.*, 2015 ▸) and the preparation of di­hydroxy­arene-substituted benzimidazoles, quinazolines and larger rings *via* cyclo­condensation of di­amines (Los *et al.*, 2012 ▸).
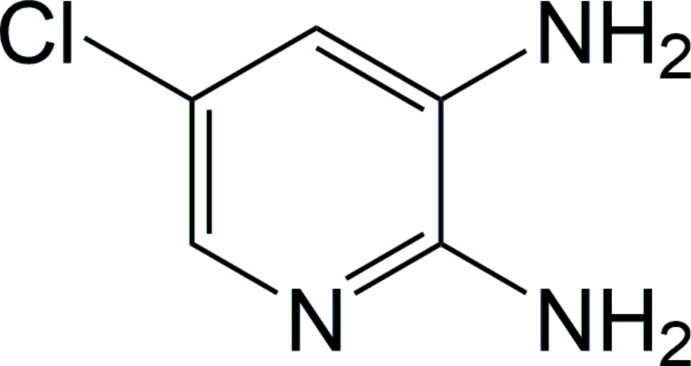


## Structural commentary   

**Figure 1:**
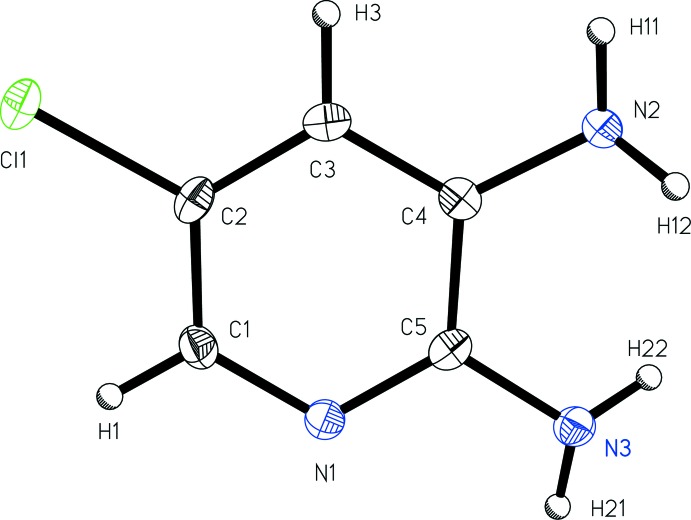
A view of 5-chloro­pyridine-2,3-di­amine (I) with the atom-numbering scheme. Displacement ellipsoids are shown at the 50% probability level.

The mol­ecular structure of the title compound 5-chloro­pyridine-2,3-di­amine (Fig. 1 ▸) shows that the mol­ecule is nearly planar with r.m.s deviation from the mean plane of all non-hydrogen atoms of 0.013 (3) Å. The amino groups *ortho* and *meta* to the pyridine nitro­gen atom twist out of the plane of the mol­ecule in such a way as to minimize contact with one another, with NH_2_ plane to mol­ecular plane angles of 45 (3) and 34 (3)° for N2 and N3, respectively. It is notable that the achiral title compound crystallizes in a non-enanti­ogenic (Söhncke) space group, although not a polar space group.

## Supra­molecular features   

**Figure 2:**
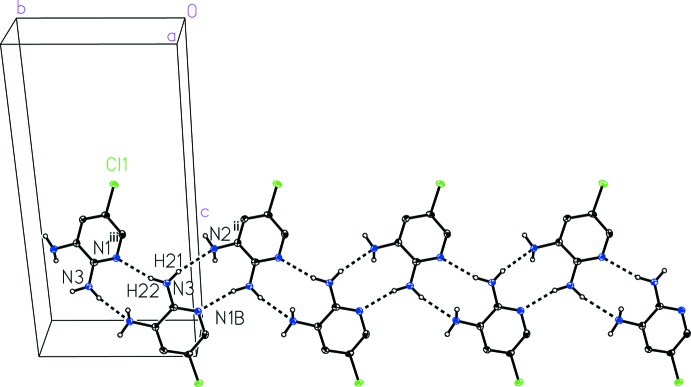
A view of the inter­molecular N3_amine_—H21⋯N2^ii^_amine_ and N3_amine_—H22⋯N1^iii^_pyr_ one-dimensional hydrogen bonding in 5-chloro­pyridine-2,3-di­amine (I). [Symmetry codes: (ii) −*x* + 2, *y* − 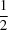, −*z* + 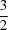; (iii) −*x* + 2, *y* + 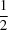, −*z* + 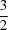.]

**Figure 3:**
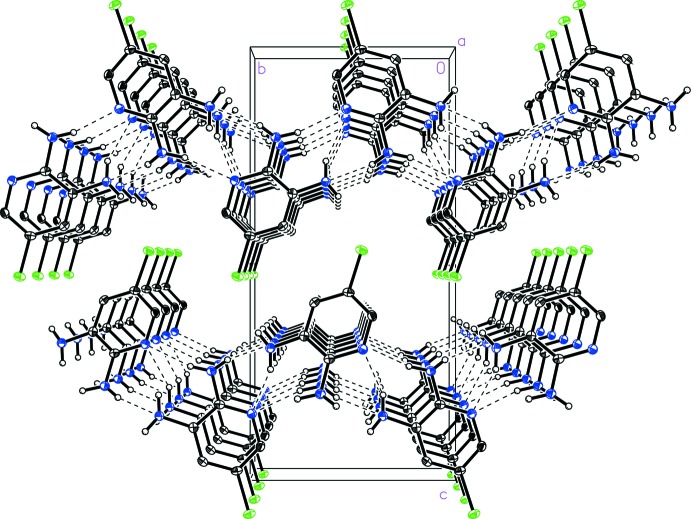
A view of the packing in 5-chloro­pyridine-2,3-di­amine (I) indicating hydrogen bonding connecting the one-dimensional strips into two-dimensional sheets along with offset face-to-face π-stacking.

**Figure 4:**
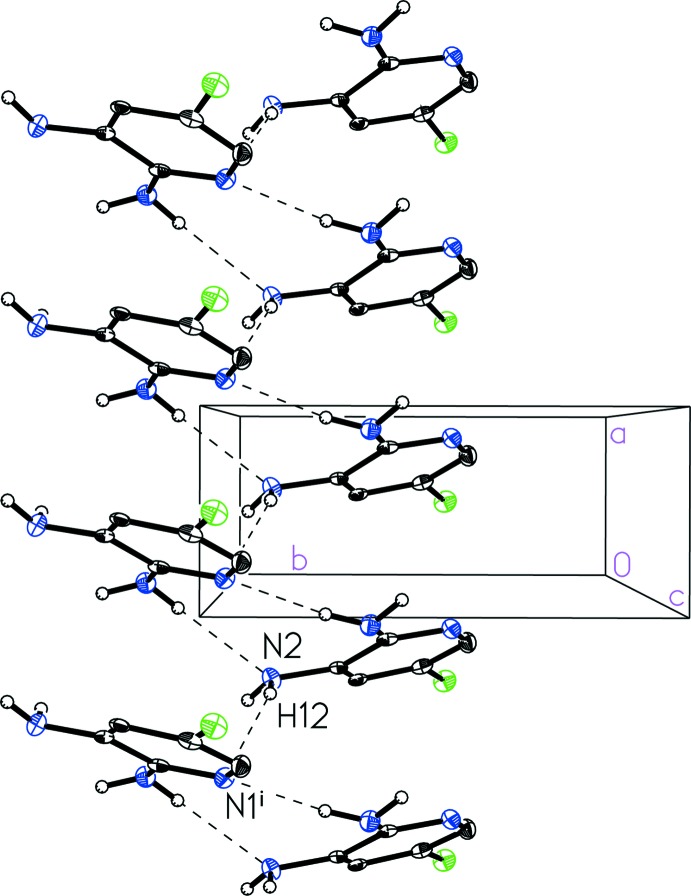
A view of the spiral hydrogen-bonded chain in 5-chloro­pyridine-2,3-di­amine (I) highlighting the N2_amine_—H12⋯N1^i^_pyr_ contact. [Symmetry code: (i) −*x* + 1, *y* + 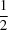, −*z* + 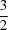.]

Notable inter­molecular inter­actions observed in the structure of 5-chloro­pyridine-2,3-di­amine (I) include N_amine_—H⋯N_pyr_ and N_amine_—H⋯N_amine_ hydrogen bonding inter­actions and offset face-to-face π-stacking. The mol­ecules connect into a one-dimensional strip running parallel to the crystallographic *b* axis (Fig. 2 ▸) with long N3_amine_—H21⋯N2^ii^_amine_ [symmetry code (ii) −*x* + 2, *y* − 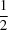, −*z* + 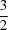] and N3_amine_—H22⋯N1^iii^_pyr_ [symmetry code (iii) −*x* + 2, *y* + 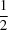, −*z* + 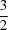] hydrogen bonding inter­actions with donor–acceptor distances of 3.250 (4) and 3.075 (4) Å, respectively (Table 1 ▸). A third N_amine_—H⋯N_pyr_ hydrogen-bonding contact and offset face-to-face π-stacking can be seen to extend along the crystallographic *a* axis (Fig. 3 ▸), acting to link the one-dimensional strips into two-dimensional sheets. The N2_amine_—H12⋯N1_pyr_^i^ [symmetry code (i) −*x* + 1, *y* + 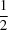, −*z* + 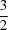] contact exhibits a donor–acceptor distance 3.264 (3) Å. The π-stacking is characterized by a centroid-to-centroid distance of 3.756 (1) Å, plane-to-plane distances of 3.414 (2) Å and a ring offset of 1.568 (3) Å (Hunter & Saunders, 1990 ▸; Lueckheide *et al.*, 2013 ▸). Alternatively, the three hydrogen-bonding contacts and the π-stacking taken together can be seen to form a spiral of 5-chloro­pyridine-2,3-di­amine (I) mol­ecules extending along the *a-*axis direction (Fig. 4 ▸).

## Database survey   

The Cambridge Structural Database (Groom *et al.*, 2016 ▸) contains about fifty structurally similar compounds to 5-chloro­pyridine-2,3-di­amine (I), with 2-amino-5-chloro­pyridine (AMCLPY12) (Pourayoubi *et al.*, 2007 ▸) and 2-amino-3-chloro­pyridine (URAXER) (Hu *et al.*, 2011 ▸) being the most chemically and structurally similar. The C—Cl bond length in the title compound, with distance 1.748 (3) Å, is comparable to those in 2-amino-5-chloro­pyridine (AMCLPY12) and 2-amino-3-chloro­pyridine (URAXER), with distances 1.7404 (14) and 1.735 (3) Å, respectively. The C—N_amine_ distances in the title compound, 1.406 (4) and 1.385 (4) Å, however, are somewhat longer than in 2-amino-5-chloro­pyridine (AMCLPY12) [1.3602 (19) Å] and 2-amino-3-chloro­pyridine (URAXER) [1.351 (4) Å]. 2-amino-5-chloro­pyridine (AMCLPY12), which does not have the *meta*-NH_2_ substitution of the title compound, packs in a herringbone formation featuring centrosymmetric head-to-tail N_amine_—H⋯N_pyr_ hydrogen bonding dimers with donor–acceptor distance 3.031 (2) Å. 2-Amino-3-chloro­pyridine (URAXER), has a *meta*-Cl substitution in place of the *meta*-NH_2_ in the title compound. Like 2-amino-5-chloro­pyridine (AMCLPY12), 2-amino-3-chloro­pyridine (URAXER) features a herringbone packing with centrosymmetric head-to-tail N_amine_—H⋯N_pyr_ hydrogen-bonded dimer with a similar donor–acceptor distance of 3.051 (5) Å. The similar hydrogen-bonding motif in these two related compounds differs from the title compound, which does not exhibit centrosymmetric hydrogen-bonding dimerization. 2-Amino-3-chloro­pyridine (URAXER) also has short inter­molecular Cl⋯Cl inter­actions of 3.278 (3) Å, where no such short halogen–halogen contact was observed in 2-amino-5-chloro­pyridine (AMCLPY12) or the title compound.

## Synthesis and crystallization   

5-Chloro­pyridine-2,3-di­amine (97%) was purchased from Aldrich Chemical Company, USA. A single crystal suitable for analysis was selected from the purchased sample and used as received.

## Analytical data   

^1^H NMR (Bruker Avance 400 MHz, DMSO *d*_6_): δ 4.99 (*br s*, 2 H, N*H*_2_), 5.55 (*br s*, 2 H, N*H*_2_), 6.69 (*d*, 1 H, *J* = 2.3 Hz, C_ar­yl_ *H*), 7.21 (*d*, 1 H, *J* = 2.3 Hz, C_ar­yl_ *H*). ^13^C NMR (^13^C{^1^H}, 100.6 MHz, DMSO *d*_6_): δ 116.58 (*C*_ar­yl_H), 118.38 (*C*_ar­yl_), 131.32 (*C*_ar­yl_), 131.66 (*C*_ar­yl_H), 147.10 (*C*_ar­yl_). IR (Thermo Nicolet iS50, ATR, cm^−1^): 3392 (*m*, N—H *str*), 3309 (*m*, N—H *str*), 3172 (*m*, aryl C—H *str*), 1637 (*s*, aryl C=C *str*), 1572 (*m*), 1472 (*s*), 1421 (*m*), 1347 (*w*), 1307 (*w*), 1280 (*w*), 1240 (*m*), 1068 (*m*), 939 (*w*), 887 (*w*), 861 (*m*), 792 (*m*), 770 (*m*), 680 (*s*), 630 (*s*), 568 (*s*), 490 (*s*), 449 (*s*). GC/MS (Hewlett-Packard MS 5975/GC 7890): *M* ^+^ = 143 (calc. exact mass = 143.03).

## Refinement   

Crystal data, data collection and structure refinement details are summarized in Table 2 ▸. All non-hydrogen atoms were refined anisotropically. Hydrogen atoms on carbon were included in calculated positions and refined using a riding model with C—H = 0.95 Å and *U*_iso_(H) = 1.2*U*_eq_(C) of the aryl C-atoms. The positions of the four amino hydrogen atoms were found in the difference map and they were refined semi-freely using a distance restraint *d*(N—H) = 0.91 Å, and *U*_iso_(H) = 1.2*U*_eq_(N).

## Supplementary Material

Crystal structure: contains datablock(s) global, I. DOI: 10.1107/S2056989017010489/fy2122sup1.cif

Structure factors: contains datablock(s) I. DOI: 10.1107/S2056989017010489/fy2122Isup2.hkl

Supporting information file. DOI: 10.1107/S2056989017010489/fy2122Isup3.cml

CCDC reference: 1562267

Additional supporting information:  crystallographic information; 3D view; checkCIF report

## Figures and Tables

**Table 1:** Hydrogen-bond geometry (Å, °)

*D*—H⋯*A*	*D*—H	H⋯*A*	*D*⋯*A*	*D*—H⋯*A*
N2—H12⋯N1^i^	0.86 (2)	2.48 (3)	3.264 (3)	151 (3)
N3—H21⋯N2^ii^	0.89 (2)	2.38 (2)	3.250 (4)	166 (3)
N3—H22⋯N1^iii^	0.90 (2)	2.19 (2)	3.075 (4)	167 (3)

Symmetry codes: (i) 
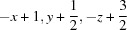
; (ii) 
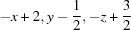
; (iii) 
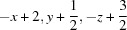
.

**Table 2:** Experimental details

Crystal data	
Chemical formula	C_5_H_6_ClN_3_
*M*_r_	143.58
Crystal system, space group	Orthorhombic, *P*2_1_2_1_2_1_
Temperature (K)	125
*a*, *b*, *c* (Å)	3.7565 (8), 8.7002 (17), 18.350 (4)
*V* (Å^3^)	599.7 (2)
*Z*	4
Radiation type	Mo *K*α
μ (mm^−1^)	0.53
Crystal size (mm)	0.10 × 0.05 × 0.04

Data collection	
Diffractometer	Bruker APEXII CCD
Absorption correction	Multi-scan (*SADABS*; Bruker, 2013 ▸)
*T*_min_, *T*_max_	0.75, 0.98
No. of measured, independent and observed [*I* > 2σ(*I*)] reflections	14989, 1845, 1477
*R*_int_	0.087
(sin θ/λ)_max_ (Å^−1^)	0.714

Refinement	
*R*[*F* ^2^ > 2σ(*F* ^2^)], *wR*(*F* ^2^), *S*	0.041, 0.084, 1.07
No. of reflections	1845
No. of parameters	94
No. of restraints	4
H-atom treatment	H atoms treated by a mixture of independent and constrained refinement
Δρ_max_, Δρ_min_ (e Å^−3^)	0.37, −0.38
Absolute structure	Flack *x* determined using 511 quotients [(*I* ^+^)−(*I* ^−^)]/[(*I* ^+^)+(*I* ^−^)] (Parsons *et al.*, 2013 ▸)
Absolute structure parameter	0.01 (6)

Computer programs: *APEX2* and *SAINT* (Bruker, 2013 ▸), *SHELXT2014* (Sheldrick, 2015*a* ▸), *SHELXL2014* (Sheldrick, 2015*b* ▸), *SHELXTL2014* (Sheldrick, 2008 ▸), *OLEX2* (Dolomanov *et al.*, 2009 ▸) and *Mercury* (Macrae *et al.*, 2008 ▸).

## supplementary crystallographic information

### Crystal data

**Table:**

C_5_H_6_ClN_3_	*D*_x_ = 1.590 Mg m^−^^3^
*M**_r_* = 143.58	Mo *K*α radiation, λ = 0.71073 Å
Orthorhombic, *P*2_1_2_1_2_1_	Cell parameters from 4306 reflections
*a* = 3.7565 (8) Å	θ = 2.6–29.7°
*b* = 8.7002 (17) Å	µ = 0.53 mm^−^^1^
*c* = 18.350 (4) Å	*T* = 125 K
*V* = 599.7 (2) Å^3^	Plate, colourless
*Z* = 4	0.10 × 0.05 × 0.04 mm
*F*(000) = 296	

### Data collection

**Table:**

Bruker APEXII CCD diffractometer	1845 independent reflections
Radiation source: fine-focus sealed tube	1477 reflections with *I* > 2σ(*I*)
Graphite monochromator	*R*_int_ = 0.087
Detector resolution: 8.3333 pixels mm^-1^	θ_max_ = 30.5°, θ_min_ = 2.2°
φ and ω scans	*h* = −5→5
Absorption correction: multi-scan (SADABS; Bruker, 2013)	*k* = −12→12
*T*_min_ = 0.75, *T*_max_ = 0.98	*l* = −26→26
14989 measured reflections	

### Refinement

**Table:**

Refinement on *F*^2^	Hydrogen site location: mixed
Least-squares matrix: full	H atoms treated by a mixture of independent and constrained refinement
*R*[*F*^2^ > 2σ(*F*^2^)] = 0.041	*w* = 1/[σ^2^(*F*_o_^2^) + (0.0308*P*)^2^ + 0.2158*P*] where *P* = (*F*_o_^2^ + 2*F*_c_^2^)/3
*wR*(*F*^2^) = 0.084	(Δ/σ)_max_ < 0.001
*S* = 1.07	Δρ_max_ = 0.37 e Å^−^^3^
1845 reflections	Δρ_min_ = −0.38 e Å^−^^3^
94 parameters	Absolute structure: Flack *x* determined using 511 quotients [(*I*^+^)-(*I*^-^)]/[(*I*^+^)+(*I*^-^)] (Parsons *et al.*, 2013)
4 restraints	Absolute structure parameter: 0.01 (6)

### Special details

**Table:**

Geometry. All esds (except the esd in the dihedral angle between two l.s. planes) are estimated using the full covariance matrix. The cell esds are taken into account individually in the estimation of esds in distances, angles and torsion angles; correlations between esds in cell parameters are only used when they are defined by crystal symmetry. An approximate (isotropic) treatment of cell esds is used for estimating esds involving l.s. planes.

### Fractional atomic coordinates and isotropic or equivalent isotropic displacement parameters (Å^2^)

**Table:**

	*x*	*y*	*z*	*U*_iso_*/*U*_eq_	
Cl1	0.5035 (2)	0.46707 (8)	0.47194 (3)	0.01872 (16)	
N1	0.8538 (6)	0.4647 (3)	0.67658 (12)	0.0148 (5)	
N2	0.5894 (7)	0.8717 (3)	0.67694 (14)	0.0153 (5)	
H11	0.464 (9)	0.928 (3)	0.6466 (14)	0.018*	
H12	0.530 (10)	0.872 (3)	0.7224 (12)	0.018*	
N3	0.8976 (7)	0.6490 (3)	0.76777 (13)	0.0154 (5)	
H21	1.032 (9)	0.580 (3)	0.7905 (15)	0.018*	
H22	0.955 (10)	0.748 (3)	0.7772 (16)	0.018*	
C1	0.7608 (8)	0.4231 (3)	0.60820 (16)	0.0157 (6)	
H1	0.7977	0.3199	0.5931	0.019*	
C2	0.6148 (7)	0.5258 (4)	0.56011 (14)	0.0138 (5)	
C3	0.5497 (7)	0.6759 (3)	0.58152 (14)	0.0121 (6)	
H3	0.445	0.747	0.5486	0.015*	
C4	0.6388 (7)	0.7209 (3)	0.65122 (15)	0.0122 (6)	
C5	0.8035 (7)	0.6100 (3)	0.69716 (15)	0.0125 (6)	

### Atomic displacement parameters (Å^2^)

**Table:**

	*U*^11^	*U*^22^	*U*^33^	*U*^12^	*U*^13^	*U*^23^
Cl1	0.0204 (3)	0.0220 (3)	0.0138 (3)	−0.0021 (4)	−0.0034 (3)	−0.0035 (3)
N1	0.0154 (11)	0.0144 (11)	0.0145 (11)	0.0014 (11)	−0.0007 (9)	−0.0001 (11)
N2	0.0182 (14)	0.0137 (12)	0.0140 (11)	0.0036 (9)	−0.0003 (9)	0.0000 (9)
N3	0.0162 (14)	0.0152 (12)	0.0148 (11)	−0.0009 (10)	−0.0036 (9)	0.0004 (10)
C1	0.0176 (15)	0.0126 (14)	0.0171 (14)	0.0008 (11)	0.0014 (12)	−0.0019 (11)
C2	0.0101 (12)	0.0202 (13)	0.0112 (11)	−0.0036 (12)	0.0002 (9)	−0.0016 (12)
C3	0.0061 (14)	0.0157 (12)	0.0146 (12)	−0.0022 (10)	0.0009 (10)	0.0040 (10)
C4	0.0064 (12)	0.0142 (14)	0.0160 (14)	−0.0003 (10)	0.0026 (11)	0.0004 (11)
C5	0.0066 (13)	0.0184 (15)	0.0125 (13)	−0.0017 (11)	0.0021 (10)	0.0011 (11)

### Geometric parameters (Å, º)

**Table:**

Cl1—C2	1.748 (3)	N3—H22	0.90 (2)
N1—C5	1.333 (4)	C1—C2	1.371 (4)
N1—C1	1.352 (4)	C1—H1	0.95
N2—C4	1.406 (4)	C2—C3	1.385 (4)
N2—H11	0.88 (2)	C3—C4	1.379 (4)
N2—H12	0.86 (2)	C3—H3	0.95
N3—C5	1.385 (4)	C4—C5	1.423 (4)
N3—H21	0.89 (2)		
			
C5—N1—C1	118.7 (3)	C1—C2—Cl1	120.1 (2)
C4—N2—H11	112 (2)	C3—C2—Cl1	119.7 (2)
C4—N2—H12	111 (2)	C4—C3—C2	119.2 (3)
H11—N2—H12	118 (3)	C4—C3—H3	120.4
C5—N3—H21	115 (2)	C2—C3—H3	120.4
C5—N3—H22	118 (2)	C3—C4—N2	123.0 (3)
H21—N3—H22	114 (3)	C3—C4—C5	117.5 (3)
N1—C1—C2	121.8 (3)	N2—C4—C5	119.4 (3)
N1—C1—H1	119.1	N1—C5—N3	117.5 (3)
C2—C1—H1	119.1	N1—C5—C4	122.5 (2)
C1—C2—C3	120.2 (3)	N3—C5—C4	119.9 (3)
			
C5—N1—C1—C2	−0.5 (4)	C1—N1—C5—N3	179.3 (2)
N1—C1—C2—C3	−1.7 (4)	C1—N1—C5—C4	3.3 (4)
N1—C1—C2—Cl1	179.3 (2)	C3—C4—C5—N1	−4.0 (4)
C1—C2—C3—C4	1.0 (4)	N2—C4—C5—N1	179.0 (3)
Cl1—C2—C3—C4	−180.0 (2)	C3—C4—C5—N3	−179.8 (3)
C2—C3—C4—N2	178.5 (3)	N2—C4—C5—N3	3.2 (4)
C2—C3—C4—C5	1.7 (4)		

### Hydrogen-bond geometry (Å, º)

**Table:**

*D*—H···*A*	*D*—H	H···*A*	*D*···*A*	*D*—H···*A*
N2—H12···N1^i^	0.86 (2)	2.48 (3)	3.264 (3)	151 (3)
N3—H21···N2^ii^	0.89 (2)	2.38 (2)	3.250 (4)	166 (3)
N3—H22···N1^iii^	0.90 (2)	2.19 (2)	3.075 (4)	167 (3)

Symmetry codes: (i) −*x*+1, *y*+1/2, −*z*+3/2; (ii) −*x*+2, *y*−1/2, −*z*+3/2; (iii) −*x*+2, *y*+1/2, −*z*+3/2.

## References

[bb1] Baladi, T., Granzhan, A. & Piguel, S. (2016). *Eur. J. Org. Chem.* pp. 2421–2434.

[bb2] Bruker (2013). *SAINT*, *SADABS* and *APEX2*. Bruxer AXS Inc., Madison, Wisconsin, USA.

[bb3] Dolomanov, O. V., Bourhis, L. J., Gildea, R. J., Howard, J. A. K. & Puschmann, H. (2009). *J. Appl. Cryst.* **42**, 339–341.

[bb4] Groom, C. R., Bruno, I. J., Lightfoot, M. P. & Ward, S. C. (2016). *Acta Cryst.* B**72**, 171–179. 10.1107/S2052520616003954 PMC4822653 27048719

[bb5] Han, Z., Hao, X., Ma, B. & Zhu, C. (2016). *Eur. J. Med. Chem.* **121**, 308–317. 10.1016/j.ejmech.2016.05.036 27267001

[bb6] Hu, Z.-N., Yang, H.-B., Luo, H. & Li, B. (2011). *Acta Cryst.* E**67**, o1138. 10.1107/S1600536811013432 PMC3089079 21754447

[bb7] Hunter, C. A. & Sanders, J. K. M. (1990). *J. Am. Chem. Soc.* **112**, 5525–5534.

[bb8] Israel, M. & Day, A. R. (1959). *J. Org. Chem.* **24**, 1455–1460.

[bb9] Los, R., Wesołowska-Trojanowska, M., Malm, A., Karpińska, M. M., Matysiak, J., Niewiadomy, A. & Głaszcz, U. (2012). *Heteroat. Chem.* **23**, 265–275.

[bb10] Lueckheide, M., Rothman, N., Ko, B. & Tanski, J. M. (2013). *Polyhedron*, **58**, 79–84.

[bb11] Macrae, C. F., Bruno, I. J., Chisholm, J. A., Edgington, P. R., McCabe, P., Pidcock, E., Rodriguez-Monge, L., Taylor, R., van de Streek, J. & Wood, P. A. (2008). *J. Appl. Cryst.* **41**, 466–470.

[bb12] Parsons, S., Flack, H. D. & Wagner, T. (2013). *Acta Cryst.* B**69**, 249–259. 10.1107/S2052519213010014 PMC3661305 23719469

[bb13] Pourayoubi, M., Ghadimi, S. & Ebrahimi Valmoozi, A. A. (2007). *Acta Cryst.* E**63**, o4631. 10.1107/S1600536810002692 PMC2979840 21579865

[bb14] Püsküllü, M. O., Karaaslan, C., Bakar, F. & Göker, H. (2015). *Chem. Heterocycl. Compd.* **51**, 723–733.

[bb15] Sheldrick, G. M. (2008). *Acta Cryst.* A**64**, 112–122. 10.1107/S0108767307043930 18156677

[bb16] Sheldrick, G. M. (2015*a*). *Acta Cryst.* A**71**, 3–8.

[bb17] Sheldrick, G. M. (2015*b*). *Acta Cryst.* C**71**, 3–8.

[bb18] Taha, M., Ismail, N. H., Imran, S., Rashwan, H., Jamil, W., Ali, S., Kashif, S. M., Rahim, F., Salar, U. & Khan, K. M. (2016). *Bioorg. Chem.* **65**, 48–56. 10.1016/j.bioorg.2016.01.007 26855413

[bb19] Xie, D., Lu, J., Xie, J., Cui, J., Li, T.-F., Wang, Y.-C., Chen, Y., Gong, N., Li, X.-Y., Fu, L. & Wang, Y.-X. (2016). *Eur. J. Med. Chem.* **117**, 19–32. 10.1016/j.ejmech.2016.04.017 27089209

